# Understanding the Mechanism of Recognition of Gab2 by the N-SH2 Domain of SHP2

**DOI:** 10.3390/life10060085

**Published:** 2020-06-11

**Authors:** Lorenzo Visconti, Francesca Malagrinò, Livia Pagano, Angelo Toto

**Affiliations:** Istituto Pasteur—Fondazione Cenci Bolognetti, Dipartimento di Scienze Biochimiche “A. Rossi Fanelli” and Istituto di Biologia e Patologia Molecolari del CNR, Sapienza Università di Roma, 00185 Rome, Italy; lorenzo.visconti@uniroma1.it (L.V.); francesca.malagrino@uniroma1.it (F.M.); livia.pagano@uniroma1.it (L.P.)

**Keywords:** binding kinetics, intrinsically disordered protein, mutagenesis

## Abstract

Gab2 is a scaffold protein with a crucial role in colocalizing signaling proteins and it is involved in the regulation of several important molecular pathways. SHP2 is a protein phosphatase that binds, through its two SH2 domains, specific consensus sequences presenting a phosphorylated tyrosine located on the disordered tail of Gab2. To shed light on the details of such a fundamental interaction for the physiology of the cell, we present a complete mutational analysis of the kinetics of binding between the N-SH2 domain of SHP2 and a peptide mimicking a specific region of Gab2. By analyzing kinetic data, we determined structural features of the transition state of the N-SH2 domain binding to Gab2, highlighting a remarkable cooperativity of the binding reaction. Furthermore, comparison of these data with ones previously obtained for another SH2 domain suggests the presence of underlying general features characterizing the binding process of SH2 domains. Data are discussed under the light of previous works on SH2 domains.

## 1. Introduction

It is general knowledge that the complexity of an organism is not directly correlated with the size of its genome and the number of its genes. Evolution is more often the result of the arising of novel intracellular interactions, rather than the acquisition of new genes and, consequently, of completely new proteins. Thus, with the increase in complexity of eukaryotic organisms during evolution, intracellular milieu has become progressively more intricate and increasingly finely regulated. Thousands of proteins simultaneously recognizing different ligands are at the basis of the most fundamental physiological pathways that regulate cellular life. Frequently, the interactions between proteins are mediated by specific classes of domains that usually recognize a determined consensus sequence [1,2,3,4,5]. To avoid the misregulation of cell physiology, different protein–protein interaction domains evolved to ensure specificity and proper affinity for their natural ligand(s) [6].

Understanding the mechanism of how interactions between proteins occur is of fundamental importance to comprehend the basis of the regulation of the molecular pathways in which they are involved. Many protein–protein interactions are based on the recognition of short binding regions, called Short Linear Motifs, mediated by globular domain families, such as SH2, SH3 and PDZ domains [1,2,3,4,5]. Domains belonging to the same families are characterized by sharing a conserved topology and, consequently, binding specificity. In fact, recognition of ligands occurs at the level of well-established consensus sequences, conserved in the domain families, rather than strings of specific amino acids. Under this light, the evidences of complicated mechanisms of binding characterized by the presence of fine allosteric networks are not surprising [7,8,9,10]. Long allosteric networks regulate the affinity and specificity for ligand(s) by a harmonized involvement of different regions of the globular domain, in the absence of major structural rearrangements [11,12].

Scaffold proteins play a fundamental role in the spatial and temporal regulation of several molecular pathways by sequestering signaling proteins in specific subcellular compartments. To do so, they typically present multiple modular interaction motifs mediating the assembly of different signaling machineries [13]. Gab2 is a scaffold protein that is structurally composed by a Pleckstrin Homology (PH) domain, that anchors the protein to the plasma membrane of the cell, and a C-terminal long disordered region presenting several binding sites for signaling proteins [14,15]. Many of these interacting motifs undergo phosphorylation of specific tyrosine residues [16,17,18], so that they can be recognized by signaling proteins containing SH2 domains, such as Grb2, PI3K and SHP2.

SHP2 is a phosphatase protein that regulates several important physiological pathways [19]. From a structural perspective, it is composed of two adjacent SH2 domains (N-SH2 and C-SH2) followed by a PTP (Protein-Tyrosine Phosphatase) domain which retains catalytic phosphatase activity [20]. SH2 domains are a class of domains of about 100 amino acids that mediate protein–protein interactions in a wide range of multidomain proteins (REF). Their topology is conserved and is characterized by the presence of a β-sheet, composed of three to five anti-parallel β-strands, flanked by two α-helices [21,22]. SH2 domains have the biological role to recognize consensus sequences characterized by the presence of a phosphorylated tyrosine (pY) together with a series of additional residues at the C-terminus of the pY [23,24]. The two SH2 domains of SHP2 mediate the interaction of the phosphatase with different partners in the intracellular environment. Interestingly, the N-SH2 domain possesses a regulatory role in the activity of the PTP domain by acting as a conformational switch [20]. In the inactive form of SHP2, the catalytic active site of the PTP domain is physically blocked by the N-SH2 domain. When the latter recognizes and binds a ligand, it undergoes a major conformational change removing the autoinhibition and triggering the activation of the phosphatase activity [20]. 

Comparison of the binding reactions of domains sharing the same topology but a different primary structure with their natural ligands is an effective tool to depict subtle and elusive processes acting in the binding event. In this paper, we provide a complete characterization, through a combination of mutagenesis and kinetics, of the binding reaction occurring between the N-SH2 domain of SHP2 and a peptide mimicking a specific disordered region of Gab2 and ranging from residue 608 to 620. The analysis of kinetic data allowed us to determine structural features of the transition state of the N-SH2 domain binding to Gab2, revealing a remarkable cooperativity of the binding reaction. In analogy to protein folding studies, where the comparison of folding reactions between proteins sharing the same topology but different sequences has been demonstrated to be a powerful methodology to depict the determinants of folding mechanisms [25], we resorted to comparing results with the ones recently obtained for the binding of the N-SH2 domain of PI3K with Gab2 [10]. Interestingly, our data indicate that the two SH2 domains present similar mechanisms of binding with different specific portions of Gab2 which may possibly represent a general feature characterizing the SH2 domains class. Results are discussed under the light of previous works on SH2 domains.

## 2. Materials and Methods

### 2.1. Expression and Purification of the N-SH2 Site-Directed Variants of SHP2

N-SH2 wild-type and all the site-directed mutants were expressed and purified as described previously [26]. Site-directed mutagenesis was performed using the QuikChange mutagenesis kit (Stratagene) according to the manufacturer’s instructions.

### 2.2. Stopped-Flow Kinetic Binding and Displacement Experiments

Kinetic binding experiments were performed on a single-mixing SX-18 stopped-flow instrument (Applied Photophysics) in pseudo-first order conditions, by mixing a constant concentration (1 μM) of Gab2_608–620_ dansylated on its N-terminus versus increasing concentrations of N-SH2, ranging from 2 to 12 μM, in buffer TrisHCl 50 mM, NaCl 300 mM, pH 7.2, at 10 °C. The excitation wavelength was 280 nm, and fluorescence was collected using a 455 nm cut-off filter. At least five independent acquisitions were collected and averaged for each experiment. The resulting averages were all satisfactorily fitted with a single exponential equation.

Displacement kinetic experiments were performed by mixing a preincubated complex of N-SH2 and dansylated Gab2_608–620_ at a 1:1 stoichiometric ratio versus a high excess of non-dansylated Gab2_608–620_. Experiments were performed in the same conditions as the binding experiments. Displacement traces were fitted with a single exponential equation.

## 3. Results and Discussion

### 3.1. Mutational Analysis of the Kinetics of Binding between the N-SH2 of SHP2 and Gab2

**Figure 1:**
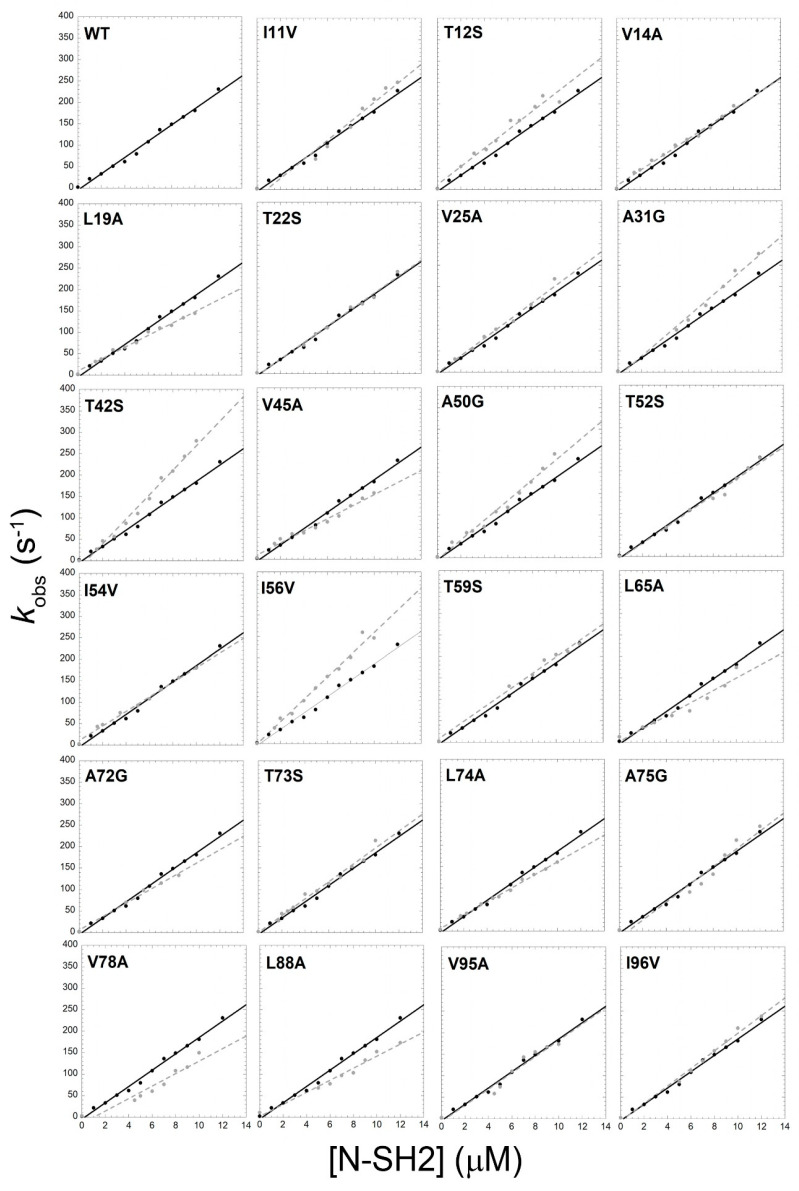
Dependences of the observed rate constants obtained at different concentrations of wild-type (black dots) and site-directed variants (gray dots) of N-SH2 through pseudo-first order binding kinetics. Lines represent the best fit to a linear function.

A powerful methodology to infer the details of a binding reaction is to perturb the system by mutating single residues and monitoring the effect of mutations on the microscopic association and dissociation rate constants. Thus, by following the same rationale used for the N-SH2 folding-value analysis [27], we resorted to producing 23 conservative site-directed variants of the N-SH2 domain of SHP2 and we monitored the binding reaction with a peptide mimicking a specific region of Gab2, ranging from residue 608 to 620 (Gab2_608–620_ N_TERM_-STGSVDYLALDFQ-C_TERM_). In analogy to our previous work [26], the binding reaction was followed spectroscopically by monitoring the change in the FRET (Fluorescence Resonance Energy Transfer) signal upon binding, by taking advantage of the tryptophan residue naturally occurring in the N-SH2 domain in position 6 as a donor group and a dansyl group covalently linked to the N-terminus of Gab2_608–620_ as the acceptor group. Time-resolved binding experiments were performed with a stopped-flow apparatus in pseudo-first order conditions, by rapidly mixing dansylated Gab2_608–620_ at a constant concentration of 1 μM versus increasing concentrations of the N-SH2 domain ranging from 2 to 12 μM, in buffer TrisHCl 50 mM, NaCl 300 mM, pH 7.2 at 10 °C. In total, 300 mM NaCl was added to the buffer in order to slow down the binding reaction and avoid the possible destabilizing mutations that would have resulted in binding kinetics too fast to be explored by a stopped-flow methodology [26]. All the binding traces were satisfactorily fitted with a single exponential equation. The dependences of the observed rate constants, *k*_obs_, obtained at different concentrations of the site-directed variants of N-SH2 (Figure 1) followed bimolecular kinetics. Given the pseudo-first order approximation, the microscopic association rate constant of the binding reaction (*k*_on_) was calculated as the slope of the fitting line, and the microscopic dissociation rate constant (*k*_off_) could be indirectly calculated as the y-axis intercept of the line. Although the extrapolation of *k*_off_ is in theory correct, the high experimental error often arising from this procedure demands a different approach. In analogy to classical experiments on hemoglobin [28], we performed displacement kinetic experiments by challenging a preincubated complex of the N-SH2 domain with dansyl-Gab2_608–620_ in a stoichiometric ratio of 1:1 at a fixed concentration of 1 μM versus a high excess of non-dansylated Gab2_608–620_, i.e., a species with the same affinity for N-SH2 but different optical properties in respect to dansyl-Gab2_608–620_. Displacement traces were fitted with a single exponential equation, and, in agreement with the theory, they were found to be insensitive to the concentration of the displacer. Association and dissociation rate constants, together with affinities and thermodynamic parameters, obtained for all the site-directed variants of N-SH2 are reported in Table 1.

### 3.2. N-SH2 SHP2: Gab2 Complex Is Stabilized by Weak Interactions

**Figure 2:**
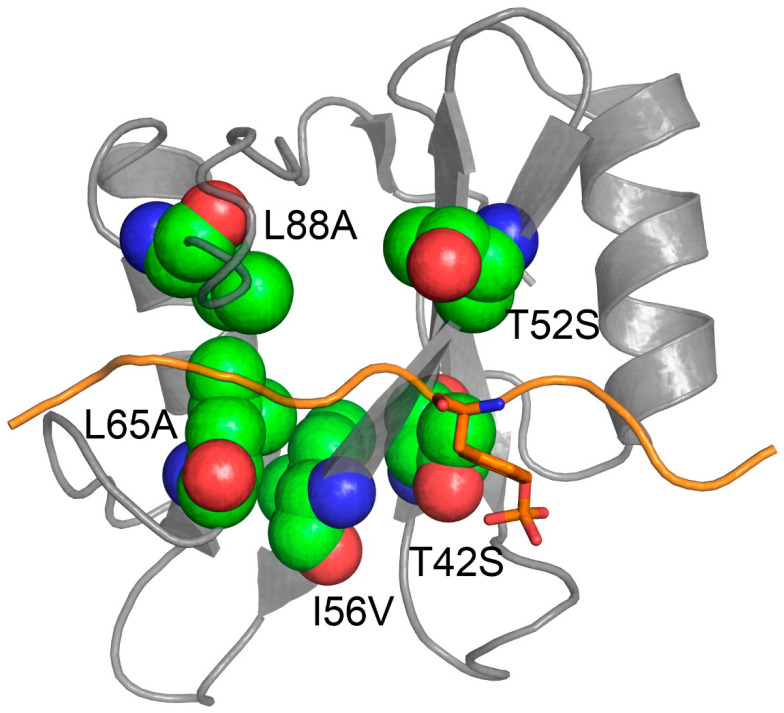
Three dimensional structure of the N-SH2 domain of SHP2 (in gray) in complex with Gab1 (in orange (Gab1 sequence: N_TERM_-GDKQVEYLDLDLD-C_TERM_ [PDB:4QSY])). Residues T42, T52, I56, L65 and L88 of N-SH2 are shown in green to highlight their physical location in respect to the binding pocket of the domain.

It is of interest to analyze the kinetic parameters associated to T42S, T52S, I56V, L65A and L88A mutations. Inspection of *K*_D_ values (calculated as *K*_D_ = *k*_off_/*k*_on_) for these variants show that T42S (*K*_D_ = 5.0 ± 0.5 nM), T52S (*K*_D_ = 10 ± 1 nM) and I56V (*K*_D_ = 30 ± 1 nM) cause a sensible increase of the affinity for Gab2_608–620_ compared to the wild-type (*K*_D_ = 100 ± 5 nM). On the other hand, L65A (*K*_D_ = 860 ± 90 nM) and L88A (*K*_D_ = 730 ± 50 nM) mutations cause a remarkable decrease in affinity. Interestingly, these effects on affinity are ascribable to an increase/decrease of the microscopic dissociation rate constant upon mutation, whilst *k*_on_ values appear to be less affected. An analysis of the structural distribution of T42, T52, I56, L65 and L88 residues reveals that they are all physically located in the binding pocket of the N-SH2 (Figure 2). Recently we characterized the effect of the T42S mutation (together with the Noonan Syndrome causing a mutation of T42A) on the binding kinetics between N-SH2 and Gab2_608–620_ [29]. A structural analysis performed by homology modelling suggested that the polar -OH group of the side chain of T42 may have the role to partially shield the negative charge of phospho-tyrosine, thus affecting the rate of release of the ligand without affecting the negative charge of the phospho-tyrosine to be in direct contact with positively charged side chains in the binding pocket. Analogously, T52S, I56V, L65A and L88A mutations do not influence the recognition of the negative charge of the phospho-tyrosine and may (de)stabilize the rate of release of the ligand through breakage/formation of weak hydrophobic and polar interactions.

### 3.3. Does a Conserved Mechanism of Binding Characterize the SH2 Domain Family?

**Figure 3:**
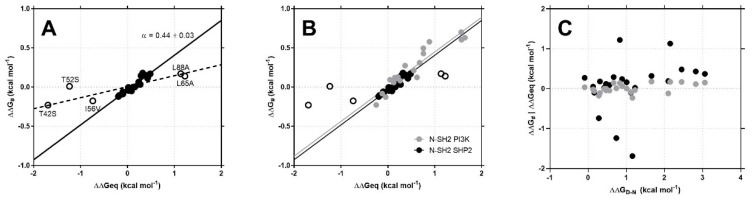
Panel **A**: Linear Free Energy Relationship (LFER) plots obtained from the analysis of kinetic data for the binding of N-SH2 domain with Gab2_608–620_. Broken line represents the best fit to a linear equation for all the points on the plot. Continuous line represents the best fit to a linear equation by excluding T42S, T52S, I56V, L65A and L88A points (details in the text). Panel **B**: Comparison of the LFER plots obtained for the N-SH2 domain of PI3K (gray line) and the N-SH2 domain of SHP2 (black line). It is evident that the two linear functions display identical slopes. Panel **C**: Plot displaying the activation free energy (ΔΔG_#_ (black dots)) and equilibrium free energy (ΔΔG_eq_ (gray dots)) values for all the site-directed variants of N-SH2 from Table 1 as a function of their thermodynamic stability (data taken from [27]).

Linear Free Energy Relationship (LFER) analysis is a powerful methodology, originally used in organic chemistry to analyze reactions involving the formation of covalent bonds, that can be used also to describe reactions involving the formation of several noncovalent interactions, such as protein folding and protein–protein interactions. Kinetic data reported in Table 1 allowed us to perform a Linear Free Energy Relationship (LFER) analysis of the binding reaction between N-SH2 and Gab2_608–620_, by relating the changes in activation free energy (DDG_#_) to the changes in equilibrium free energy (ΔΔG_eq_) of the reaction. The slope of the correlation, classically denoted as α, represents the transition state position along the reaction coordinate. The LFER plot of the binding reaction between the N-SH2 of SHP2 and Gab2 is reported in Figure 3a. A linear fit of the entire set of data was performed, returning a value of α = 0.14 ± 0.03. Despite the relatively low error arising from the fit, inspection of Figure 3a reveals that data appear to be poorly fitted. In particular, it is evident that five points corresponding to the N-SH2 variants T42S, T52S, I56V, L65A and L88A appear to be scattered and clearly deviate from the fitting line to a lower slope. By excluding the variants T42S, T52S, I56V, L65A and L88A from the analysis, the LFER reported a different α value of 0.44 ± 0.03. Importantly, the changes in activation and equilibrium free energy of binding show no correlation with the change in thermodynamic stability (ΔΔG_D-N_) of the N-SH2 variants of SHP2 (data taken from [10] (Figure 3c)).

Analogously to protein folding studies [30], the linearity of the LFER plot represents a hallmark of the cooperativity of the reaction; that is, the binding of the ligand is mediated by the entire globule and not only by residues occurring in the binding pocket. These mechanisms of interaction characterize different domain families, with the presence of allosteric networks finely regulating the affinity for ligands displaying different sequences, thus determining selectivity in the intracellular environment [8]. A powerful methodology to infer the details of the binding properties of a domain is to compare its mechanism of binding with domains sharing the same topology but displaying different sequences. Thus, it is of interest to compare the LFER analysis obtained for the N-SH2 domain of SHP2 with the one that we recently performed on the N-SH2 domain of PI3K [10]. By superimposing the LFER plots obtained for the two domains for the binding reaction with a different portion of the protein Gab2, they can be fitted with linear equation displaying identical slopes (Figure 3b).

The remarkable similarity of the LFER plots of the N-SH2 domain of SHP2 and the N-SH2 domain of PI3K reveals a similar mechanism of interaction, although the two domains display different affinities for their natural ligands [10,26]. With the exception of the SH2 domains tuning their selectivity through major structural rearrangements [31,32,33], cooperative interactions mediated by the entire domain can be at the basis of the ability of SH2 domains to adapt to ligands displaying different residues flanking the phospho-tyrosine with different specificity and affinity [34]. Further structural characterizations and double-mutant cycles kinetic experiments are demanded to quantitatively characterize the allosteric networks regulating the binding mechanism of SH2 domains.

## 4. Conclusions

Because of their abundance in the proteome and the importance in mediating and regulating fundamental molecular pathways, SH2 domains represent an important subject of study. However, the mechanisms of interaction with their natural ligands have been characterized only for a few SH2 domains. By performing an extensive mutational analysis and monitoring the effects of mutations on the binding reaction with Gab2, we showed that the N-SH2 domain of SHP2 presents a cooperative mechanism of binding involving the entire domain. The remarkable similarity of the thermodynamics of the reaction with the N-SH2 domain from PI3K suggests this mechanism may represent a common feature of SH2 domains. Further investigations will provide more information and possibly confirm this eventuality.

## Figures and Tables

**Table 1:** Kinetic and thermodynamic parameters obtained from the analysis of pseudo-first order binding kinetics between site-directed variants of N-SH2 and Gab2_608–620._

	*k*_on_ (μM^−1^ s^−1^)	*k*_off_ (s^−1^)	*K*_D_ (nM)	Activation Free Energy ∆∆G_#_ (kcal mol^−1^)	Equilibrium Free Energy ∆∆G_eq_ (kcal mol^−1^)
WT	19.0 ± 0.5	1.85 ± 0.01	100 ± 5		
I11V	22.2 ± 1.5	1.81 ± 0.01	80 ± 6	−0.09 ± 0.02	−0.10 ± 0.01
T12S	21.2 ± 1.4	1.73 ± 0.01	80 ± 6	−0.06 ± 0.01	−0.10 ± 0.02
V14A	17.9 ± 0.6	2.81 ± 0.01	160 ± 5	0.03 ± 0.01	0.27 ± 0.03
L19A	13.8 ± 0.5	2.39 ± 0.01	170 ± 6	0.18 ± 0.02	0.32 ± 0.03
T22S	19.2 ± 0.8	1.93 ± 0.01	100 ± 4	−0.01 ± 0.01	0.02 ± 0.01
V25A	20.2 ± 0.9	2.04 ± 0.07	100 ± 6	−0.04 ± 0.01	0.02 ± 0.01
A31G	23.5 ± 1.1	1.62 ± 0.01	70 ± 3	−0.12 ± 0.02	−0.19 ± 0.02
T42S	28.6 ± 1.1	0.14 ± 0.01	5.0 ± 0.5	−0.23 ± 0.02	−1.69 ± 0.10
V45A	14.1 ± 0.7	3.23 ± 0.01	230 ± 11	0.17 ± 0.10	0.48 ± 0.05
A50G	23.0 ± 1.0	1.63 ± 0.07	70 ± 4	−0.11 ± 0.01	−0.18 ± 0.02
T52S	18.7 ± 1.0	0.20 ± 0.01	10 ± 1	0.01 ± 0.01	−1.24 ± 0.10
I54V	17.0 ± 0.5	2.53 ± 0.20	150 ± 13	0.06 ± 0.03	0.24 ± 0.02
I56V	26.0 ± 1.1	0.68 ± 0.01	30 ± 1	−0.18 ± 0.02	−0.74 ± 0.07
T59S	19.1 ± 0.9	2.30 ± 0.01	120 ± 6	0.00 ± 0.01	0.12 ± 0.01
L65A	14.8 ± 1.5	12.70 ± 0.10	860 ± 90	0.14 ± 0.05	1.22 ± 0.10
A72G	14.7 ± 0.9	2.39 ± 0.01	170 ± 10	0.14 ± 0.02	0.29 ± 0.03
T73S	19.7 ± 0.8	2.09 ± 0.02	110 ± 4	−0.02 ± 0.01	0.05 ± 0.01
L74A	15.6 ± 1.9	3.23 ± 0.01	210 ± 25	0.11 ± 0.05	0.43 ± 0.04
A75G	20.7 ± 2.1	2.35 ± 0.02	110 ± 10	−0.05 ± 0.02	0.09 ± 0.01
V78A	14.5 ± 1.7	2.73 ± 0.40	190 ± 35	0.15 ± 0.04	0.37 ± 0.03
L88A	14.0 ± 0.9	10.30 ± 0.05	730 ± 50	0.17 ± 0.05	1.13 ± 0.10
V95A	18.9 ± 1.7	2.44 ± 0.01	130 ± 12	0.00 ± 0.02	0.16 ± 0.01
I96V	20.5 ± 0.8	2.06 ± 0.01	100 ± 4	−0.04 ± 0.02	0.02 ± 0.01
